# Long-Term Investigation of Retinal Function in Patients with Achromatopsia

**DOI:** 10.1167/iovs.61.11.38

**Published:** 2020-09-22

**Authors:** Michalis Georgiou, Navjit Singh, Thomas Kane, Serena Zaman, Nashila Hirji, Jonathan Aboshiha, Neruban Kumaran, Angelos Kalitzeos, Joseph Carroll, Richard G. Weleber, Michel Michaelides

**Affiliations:** 1 UCL Institute of Ophthalmology, University College London, London, United Kingdom; 2 Moorfields Eye Hospital NHS Foundation Trust, London, United Kingdom; 3 Department of Ophthalmology & Visual Sciences, Medical College of Wisconsin, Wauwatosa, Wisconsin, United States; 4 Casey Eye Institute, Oregon Health & Science University, Portland, Oregon, United States

**Keywords:** retinal phenotyping, retinal sensitivity, microperimetry, end-points, trials, inherited retinal diseases, VFMA, achromatopsia

## Abstract

**Purpose:**

To investigate the long-term natural history of retinal function of achromatopsia (ACHM).

**Methods:**

Subjects with molecularly confirmed ACHM were recruited in a prospective cohort study of mesopic microperimetry. Coefficient of repeatability and intraclass correlation coefficient (ICC) of mean sensitivity (MS) were calculated. Best-corrected visual acuity (BCVA), bivariate contour ellipse area (BCEA), contrast sensitivity (CS), MS, total volume (V_TOT_), and central field volume (V_5°_) from volumetric and topographic analyses were acquired. Correlation of functional parameters with structural findings from optical coherence tomography (OCT) was performed.

**Results:**

Eighteen subjects were recruited. Mean follow-up was 7.2 years. The MS test–retest repeatability coefficient was 1.65 decibels (dB), and the ICC was 0.973 (95% confidence interval, 0.837–0.98). Mean MS was similar for right and left eyes (16.97dB and 17.14dB, respectively). A negative significant correlation between logMAR BCVA and the retinal sensitivity indices (MS, V_TOT_, V_5°_) was found. A significant negative correlation between logCS and MS, V_TOT_, and V_5°_ was also observed. BCVA and BCEA improved during follow-up. Mean CS, MS, V_TOT_, and V_5°_ at final follow-up were similar to baseline. MS was similar between *CNGA3*- and *CNGB3*-ACHM. Patients with and without the presence of a foveal ellipsoid zone on OCT had similar MS (16.64 dB and 17.17 dB, respectively).

**Conclusions:**

We demonstrate a highly reproducible assessment of MS. Retinal function including MS, volumetric indices, and CS are stable in ACHM. Improvement of fixation stability and small changes of BCVA over time may be part of the natural history of the disease.

Achromatopsia (ACHM) is the most common cone dysfunction syndrome. It presents at either birth or early infancy with poor visual acuity, pendular nystagmus, photophobia, and loss of color vision discrimination.^1^ Disease-causing variants have been reported in *CNGA3*,^2^^,^^3^ *CNGB3*,^4^ *GNAT2*,^5^^,^^6^ *ATF6*,^7^ *PDE6H*,^8^ and *PDE6C.*^9^ *GNAT2*, *ATF6*, *PDE6H*, and *PDE6C* variants are responsible for approximately 2% of ACHM cases each.^1^^,^^10^^,^^11^ *CNGB3* and *CNGA3* are responsible for approximately 70% to 80% of cases,^12^^,^^13^ for which there are five ongoing gene therapy trials (ClinicalTrials.gov numbers NCT03758404, NCT02935517, NCT03001310, NCT02599922, and NCT02610582). The first encouraging phase I/II trial results were recently released for *CNGA3*-ACHM (NCT02610582).^14^

The presence of residual cones is critical for targeting by gene therapy intervention.^15^^,^^16^ In *CNGA3* and *CNGB3* genotypes, variable degrees of ellipsoid zone (EZ) disruption and residual cone structure have been observed.^16^^–^^20^ *GNAT2*-ACHM typically presents with a continuous EZ,^21^^,^^22^ in contrast to *PDE6C*-ACHM and *ATF6*-ACHM, for which most of the patients have no residual foveal cones.^11^^,^^23^ Evidence of structural changes over time has been suggested by some studies^24^^,^^25^ but not others.^26^^–^^28^ Optical coherence tomography (OCT) findings in a large cohort of ACHM patients (*n* = 50), with a proportional incidence-based representation of genotypes and substantial follow-up period (5.1 years), support the observation that the condition is predominantly stable in the vast majority of patients.^20^ However no functional assessment beyond visual acuity and contrast sensitivity (CS) was performed in the aforementioned study.

Investigations of retinal sensitivity and, therefore, retinal function in ACHM are limited. Genead et al.^27^ performed macular microperimetry (MP) testing (*n* = 4) and showed that the overall mean retinal sensitivity was significantly decreased compared to controls. Sundaram et al.^26^ assessed cross-sectional retinal sensitivity with mesopic MP (MP-1; Nidek Technologies, Padova, Italy) and identified a mean of 16.6 decibels (dB; *n* = 40), with a significant moderate negative correlation found between retinal sensitivity and age, best-corrected visual acuity (BCVA), and reading acuity. The same cohort was also assessed longitudinally (mean follow-up of 19 months), with no significantly different retinal sensitivity (mean, 16.5 dB).^26^ In a cross-sectional study of a well-characterized cohort of *CNGA3*-ACHM (*n* = 36), Zobor et al.^18^ observed no correlation between retinal sensitivity and age. Given the aforementioned findings, it is likely that progression in ACHM is very slow and possibly subtle. Khan et al.^29^ reported electroretinography changes in two affected adult *CNGB3* individuals after 6 and 12 years had elapsed. Long-term evaluation of retinal sensitivity has not been performed to date.

Herein, we assess cross-sectional and longitudinal MP-derived retinal sensitivity with both conventional and volumetric indices of retinal function in ACHM. We explore test–retest repeatability, interocular symmetry, genotypic variability, and the rate of progression over a long-term follow-up.

## Methods and Materials

The study was approved by the Ethics Committee of Moorfields Eye Hospital. Written informed consent and assent were obtained from all subjects as appropriate. The research followed the tenets of the Declaration of Helsinki.

### Subjects

Eighteen subjects with molecularly confirmed ACHM were recruited at a single tertiary eye hospital (Moorfields Eye Hospital, London, UK).

### Clinical Assessments

All subjects underwent a clinical history and detailed ocular examination, including BCVA using an Early Treatment Diabetic Retinopathy Study (ETDRS) chart, and CS assessment using the Pelli–Robson chart at 1 meter.

### Microperimetry

Microperimetry was performed using the Nidek Technologies MP-1 in a dark room. Pupils were dilated and cyclopleged using 2.5% phenylephrine hydrochloride solution (Bausch & Lomb Inc., Rochester, NY, USA) and 1% tropicamide ophthalmic solution (Akorn, Inc., Lake Forest, IL, USA). During each test, the non-tested contralateral eye was occluded. Fixation was monitored throughout each assessment. Patients maintained fixation by means of a 2° target. Testing was performed on a 4-apostilbs (1.27 cd/m^2^) background, which is within the mesopic range, using Goldmann stimulus size III (4 mm^2^). A variable-intensity stimulus of 200-ms duration, within the dynamic range of 0 to 20 dB, and a 4-2 testing strategy were used, with the intensity of the stimulus being reduced in 4-dB steps until the stimulus was no longer detected. The stimulus intensity then increased in 2-dB steps until detected once again. Projection of the stimulus into the blind spot at 30-second intervals tested for false-positive errors. An active eye-tracking system corrected for fixation, which helped to ensure accurate stimulus projection in relation to retinal landmarks. All subjects underwent training immediately prior to each formal testing session to ensure correct operation of the response trigger. The customized testing grid consisted of 44 testing locations and had an 8° radius to cover the macular and paramacular region. The grid pattern was of radial design with centrally condensed spacing (Supplementary Fig. S1). A mean sensitivity (MS) value was automatically computed for each test by the manufacturer's software.

Test–retest repeatability at baseline was investigated for all subjects undergoing testing twice at each visit. The test was repeated in all subjects in follow-up mode after at least 5 years had elapsed, using the same testing conditions as at the baseline assessment. Fixation stability was directly assessed during the 30 seconds prior to the start of the microperimetry using the bivariate contour ellipse area (BCEA), as reported by the Nidek software, which represents an area (in degrees) where 68% of the fixation points are located.^30^

### Volumetric Indices of Retinal Function

Perimetry data are conventionally summarized by a single global index such as MS, which is the average sensitivity value of all of the retinal locations tested. Because our test grid is radial in design and employs central condensation, we also performed three-dimensional modeling of retinal sensitivity with volumetric and topographical analyses to quantify the magnitude and extent of the visual field sensitivity.^31^^–^^34^ Topographic analysis was performed and volumetric indices were derived from microperimetry using Visual Field Modeling and Analysis (VFMA; Office of Technology Transfer & Business Development, Oregon Health & Science University, Portland, OR, USA). VFMA is a custom software application^31^ that models the hill of vision (HOV) from perimetric sensitivity data, creates visual displays, and generates volumetric indices, including the total volume (V_TOT_), which represented the entire field tested, as well as the volume of the central 5° of the field volume (V_5°_), defined by a circle centered on the fovea with a radius of 5° (Supplementary Fig. S2). The volume represents the total sensitivity across the solid angle of the base of the test grid for V_TOT_ and the entire solid angle of a 5°-radius circle selection for V_5°_; it is reported in units of decibel-steradians (dB-sr).^31^ Topographic models of the HOV with volumetric indices of sensitivity were created for all perimetry tests.

### Spectral-Domain OCT

Spectral-domain OCT (SD-OCT) imaging was performed at baseline in both eyes, following cycloplegia and pupillary dilation with tropicamide 1% and phenylephrine 2.5% eye drops. Horizontal line and volume scans were acquired with a Spectralis device (Heidelberg Engineering, Heidelberg, Germany), using the protocol employed by Sundaram et al.^26^ Qualitative assessment of foveal structure was performed by grading SD-OCT images into one of five categories as previously reported: (1) continuous EZ, (2) EZ disruption, (3) EZ absence, (4) presence of a hyporeflective zone, or (5) outer retinal atrophy.^26^ Due to the small number of patients and based on the integrity of the EZ, the patients were grouped as (1) patients with grades 1 and 2 (presence of foveal EZ), or (2) patients with grades 3, 4, and 5 (absence of foveal EZ). The presence or absence of foveal hypoplasia was also noted, defined as the persistence of one or more inner retinal layers (outer plexiform layer, inner nuclear layer, inner plexiform layer, or ganglion cell layer) through the fovea. For each subject, both right and left eyes were graded at baseline.

### Statistical Methods

Statistical analysis was carried out using SPSS Statistics 22 for Windows (IBM Corp., Armonk, NY). Significance for all statistical tests was set at *P* < 0.05. The Shapiro–Wilk test was used to test for normality for all variables. Test–retest repeatability was investigated with the Bland–Altman method. The intraclass correlation coefficient (ICC) was calculated with a two-way mixed absolute agreement model. The threshold for clinical significance for changes in BCVA was defined as a difference of ≥0.3 logMAR (≥15 ETDRS letters).^35^

## Results

### Demographics and Genetics

Eighteen subjects (eight females, 44%) were recruited, all harboring previously reported ACHM-causing variants in *CNGA3* (*n* = 10, 56%), *CNGB3* (*n* = 5, 28%), *ATF6* (*n* = 2, 11%), and *GNAT2* (*n* = 1, 6%).^11^^,^^16^^,^^20^^,^^26^^,^^28^ All subjects with *CNGB3*-ACHM harbored the variant p.Thr383Ile fs*13 in a homozygous state, which accounts for more than 70% of all *CNGB3* variants in the European population.^12^ Demographics and genetics are summarized in Supplementary Table S1.

### Test–Retest Repeatability

**Figure 1.**
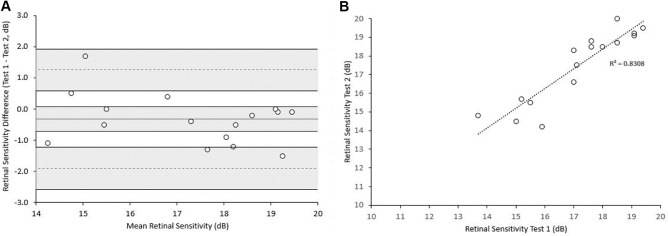
Repeatability plots. (**A**) Bland–Altman plot, assessing test–retest repeatability based on measurements on the right eyes of 16 patients at baseline. No proportional bias was observed. (**B**) Scatterplot of retinal sensitivity for the test–retest measurements.

**Figure 2.**
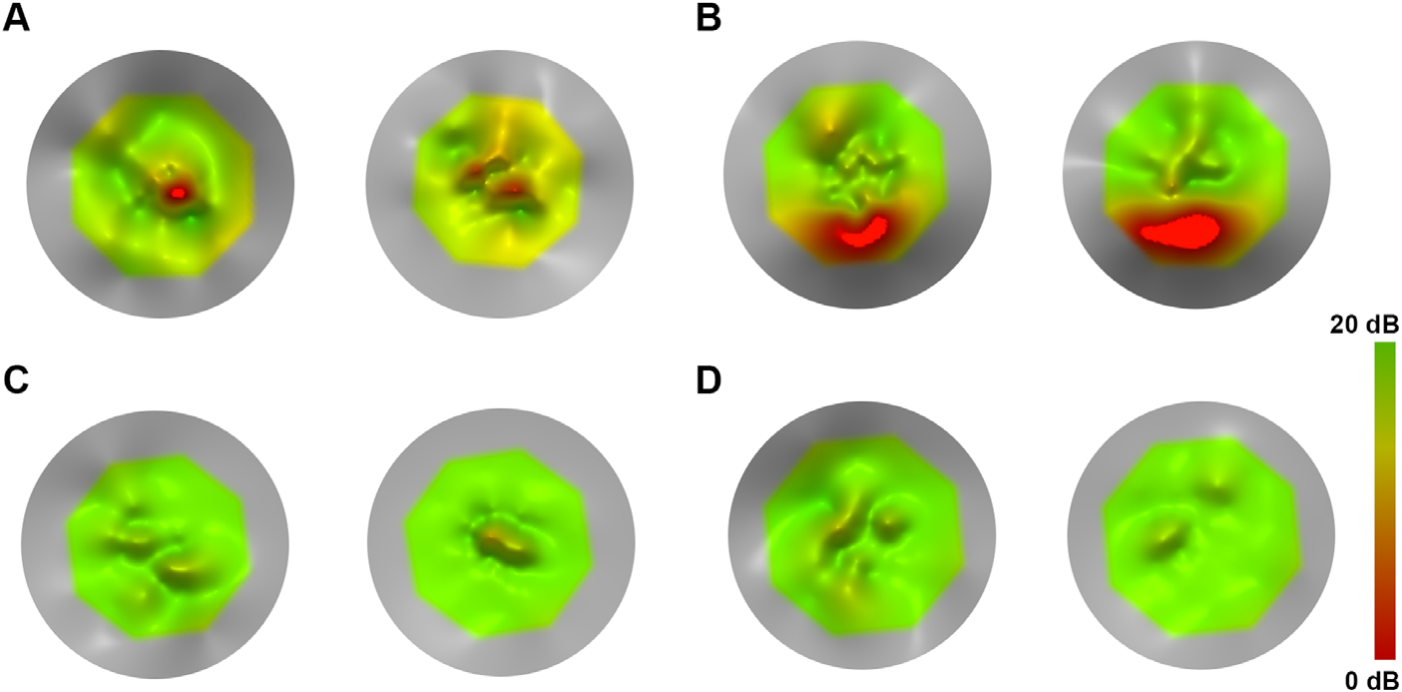
Topographical models using MP-1 microperimetry data of tests performed twice on the right eyes of four patients to assess test–retest repeatability. (**A**) A 29-year-old female with *CNGA3*-ACHM (MM_0014) showed the greatest disagreement (1.7 dB) between the two tests, with a central scotoma identified in the foveal center in both tests. (**B**) A 44-year-old male with *GNAT2*-ACHM (MM_0106) with zero difference in MS. Note that MS is the average of all tested points, so differences in topographical models (as illustrated) could occur despite the two tests having the same MS value. The patient had a scotoma in the inferior part of the plot. (**C**) A 48-year-old male with *CNGB3*-ACHM (MM_0123) with a minimal difference in MS (0.1 dB) and with one of the highest MS values in the cohort (19.45 dB). (**D**) A 23-year-old female with *ATF6*-ACHM (MM_0152) with zero difference in test–retest MS and with one of the highest MS values in the cohort (19.1 dB). The patient has no scotoma, despite the structural severity of the genotype.

Thirty-two out of 36 eyes (89%) were tested twice at baseline. To avoid a potential clustering effect, only right eyes (*n* = 16) were used to investigate test–retest repeatability. A Bland–Altman plot is presented in Figure 1A. No proportional bias was observed. The difference between a measurement and the true value would be expected to be less than 1.17 dB for 95% of observations (measurement error). The test–retest repeatability coefficient was 1.65 dB, and the difference between two measurements for the same subject was expected to be less than this for 95% of pairs of observations. The ICC was 0.973 (95% confidence interval, 0.837–0.98), indicating a high degree of agreement among tests. Figure 1B presents all the pairs of measurements. Figure 2 presents examples of test–retest repeatability.

### Disease Symmetry

**Figure 3.**
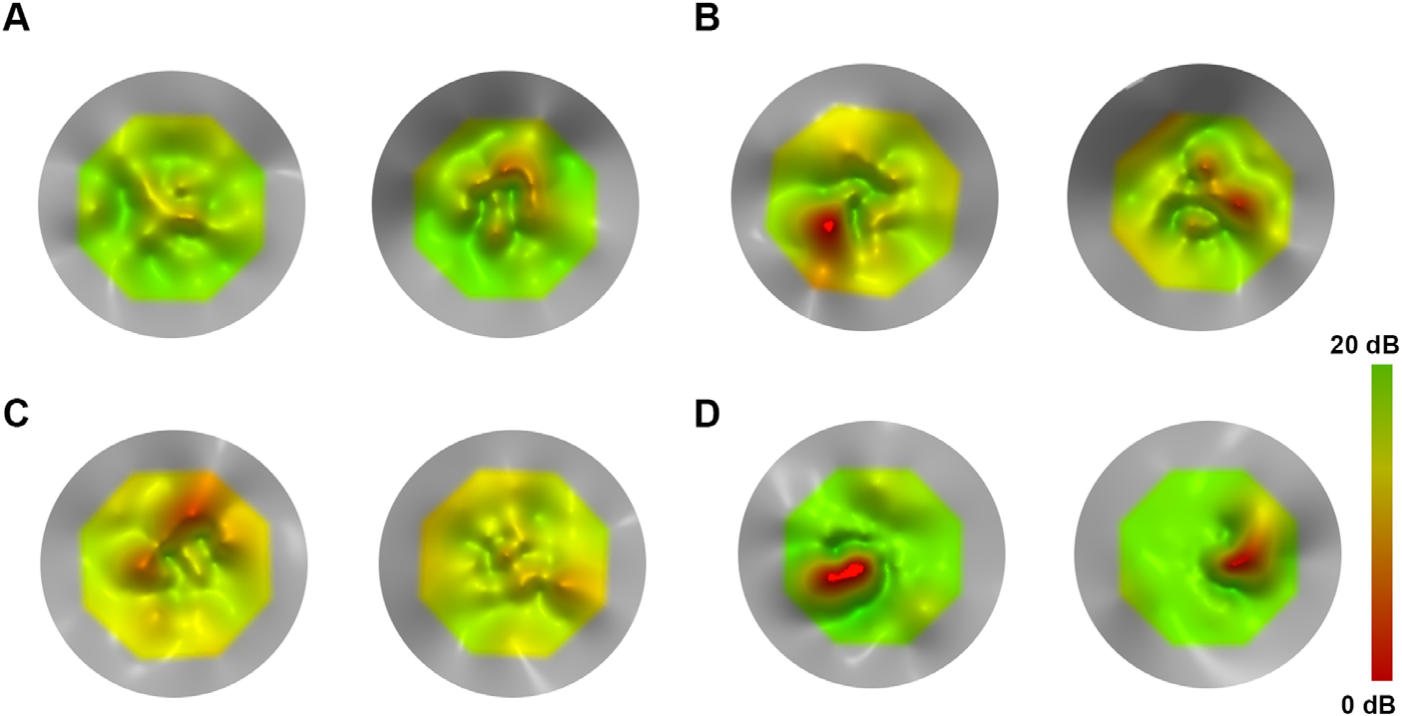
Topographical models using MP-1 microperimetry data of tests performed on the right (*left plot*) and left (*right plot*) eyes of four patients to assess disease symmetry. (**A**) A 30-year-old male with *CNGB3*-ACHM (MM_0067) with a high degree of interocular symmetry (0.3 dB MS difference). (**B**) A 7-year-old male with *CNGA3*-ACHM (MM_0165) with the greatest interocular difference in MS (3.3 dB) in the cohort. The patient undertook the test on the right eyes first (lower MS), and the difference could possibly be attributed to a learning effect and young age. (**C**) A 35-year-old male with *CNGA3*-ACHM (MM_0168) with a minimal difference in MS (0.05 dB). (**D**) An 18-year-old male with *CNGA3*-ACHM (MM_0171) with a minimal difference in MS (0.3 dB) and symmetric bilateral scotomata.

**Figure 4.**
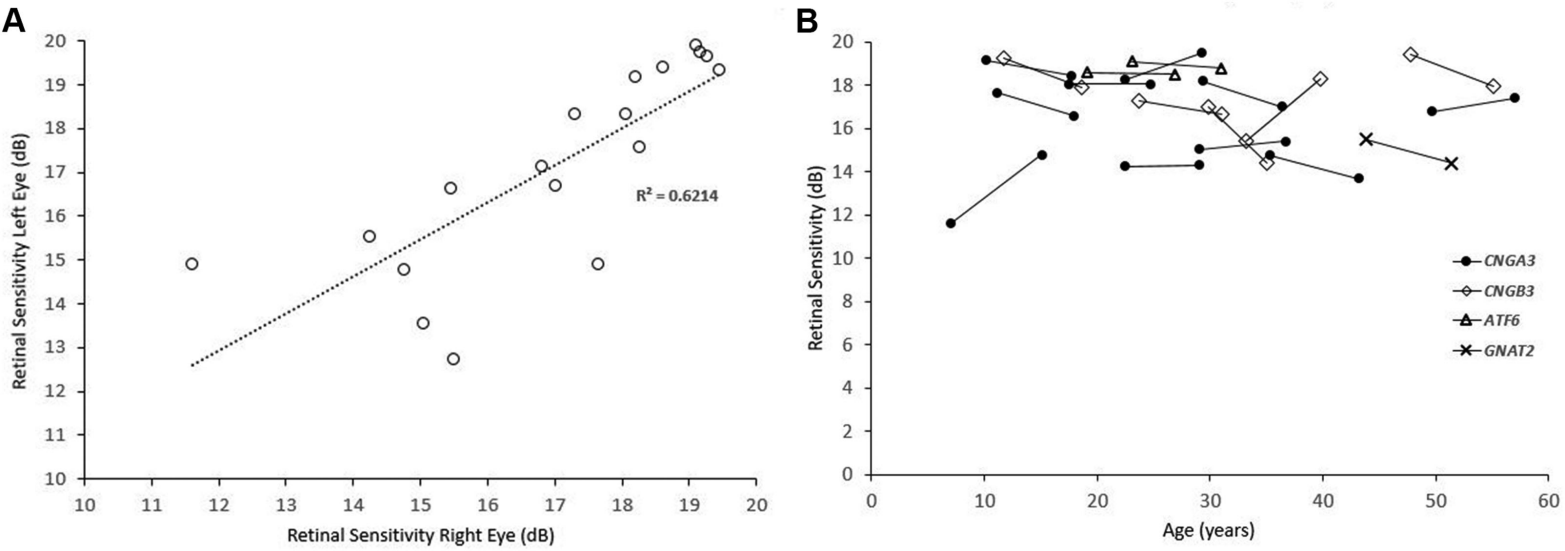
Interocular symmetry and longitudinal mean sensitivity plots. (**A**) Scatterplot of all the pairs of eyes (*n* = 18), assessed for interocular symmetry. (**B**) Plot of MS against age for each individual patient. Each genotype is plotted with a different marker.

All subjects had bilateral testing at baseline (*n* = 18). For all eyes tested twice, the mean value was used. Mean MS (SD, range) values for right and left eyes were 16.97 dB (2.08, 11.60–19.45 dB) and 17.14 dB (2.22, 12.75–19.90 dB), respectively. MS was similar in right and left eyes (paired *t*-test, *t* = –0.506, degrees of freedom [*df*] = 17, *P* = 0.62). Figure 3 presents examples of disease symmetry. The mean absolute difference (SD, range) between eyes was 1.07 dB (0.93, 0.05–3.30 dB). The subject with the greatest interocular difference was the youngest of the cohort (7 years of age at baseline) (Fig. 3C). The eye with the lower MS was tested first, and no test was repeated on either eye. Figure 4A presents MS for all pairs of eyes (*n* = 18). V_TOT_ and V_5°_ were mathematically derived from the sensitivity data and are perceived as also being symmetric. The OCT group and the presence or absence of foveal hypoplasia was the same in both eyes for all subjects.

During the follow-up visits, 16 subjects had bilateral testing and the mean absolute difference (SD, range) between eyes was 1.13 dB (1.07, 0.05–3.40 dB). Disease symmetry was sustained in follow-up, with MS being similar in right and left eyes (paired *t*-test, *t* = 0.101, *df* = 15, *P* = 0.92). With evidence of disease symmetry and in order to avoid a potential clustering effect, the baseline characteristics and the follow-up measurements were calculated using only right eyes, unless stated otherwise.

### Baseline Characteristics

Baseline characteristics are summarized in the Table. Negative statistically significant correlations between logMAR BCVA and V_TOT_ (Pearson's *r* = −0.485; *P* = 0.041) and logMAR BCVA and V_5°_ (Pearson's *r* = −0.468; *P* = 0.050) were observed (e.g., eyes with worse BCVA had lower VFMA indices). There was a weak negative association between logMAR BCVA and MS (Pearson's *r* = −0.463; *P* = 0.053). Statistically significant negative correlations between logCS and MS (Pearson's *r* = 0.552; *P* = 0.018), V_TOT_ (Pearson's *r* = 0.741; *P* < 0.001), and V_5°_ (Pearson's *r* = 0.619; *P* = 0.006) were observed (e.g., eyes with better CS had higher MS and VFMA indices). No correlation was observed between baseline age and MS (Pearson's *r* = −0.015; *P* = 0.952), V_TOT_ (Pearson's *r* = –0.236; *P* =0.346), V_5°_ (Pearson's *r* = –0.143; *P* = 0.572), BCVA (Pearson's *r* = −0.079; *P* = 0.754), or CS (Pearson's *r* = −0.236; *P* = 0.347).

**Table.** Baseline and Follow-Up Measurements

	Mean (SD, Range)	
Parameter	Baseline	Follow-Up
Age (y)	25.9 (12.3, 7–48)	33.1 (12.3, 15.1–57.0)
BCVA (logMAR)	0.89 (0.12, 0.70–1.24)	0.83 (0.11, 0.70–1.04)^*^
CS (logCS)	1.25 (0.16, 0.95–1.55)	1.21 (0.20, 0.85–1.50)
MS (dB)	16.97 (2.08, 11.60–19.45)	16.78 (1.78, 13.7–19.50)
V_TOT_ (dB-sr)	1.14 (0.19, 0.89–1.58)	1.10 (0.27, 0.56–1.77)
V_5°_ (dB-sr)	0.41 (0.04, 0.33–0.46)	0.40 (0.05, 0.32–0.47)
BCEA (deg)	13.67 (10.71, 1.63–48.5)	8.44 (5.81, 1.94–21.53)^*^

* BCVA and BCEA were the only measurements significantly different from baseline (*P* < 0.05).

### Disease Natural History

Mean follow-up (SD, range) was 7.2 years (0.6, 5.3–8.1 years). Follow-up measurements are summarized in the Table. BCVA was statistically significantly better when compared with the baseline measurement (paired *t*-test, *t* = 3.955, *df* = 17, *P* = 0.001). The mean BCVA (SD, range) absolute difference was 0.08 logMAR (0.05, 1.0–0.2 logMAR), and no patient had a clinically significant change in BCVA.^35^ Mean CS was similar to baseline (paired *t*-test, *t* = 1.154, *df* = 17, *P* = 0.265). The mean CS (SD, range) absolute difference was 0.11 logCS (0.09, 0.0–0.3 logCS).

**Figure 5.**
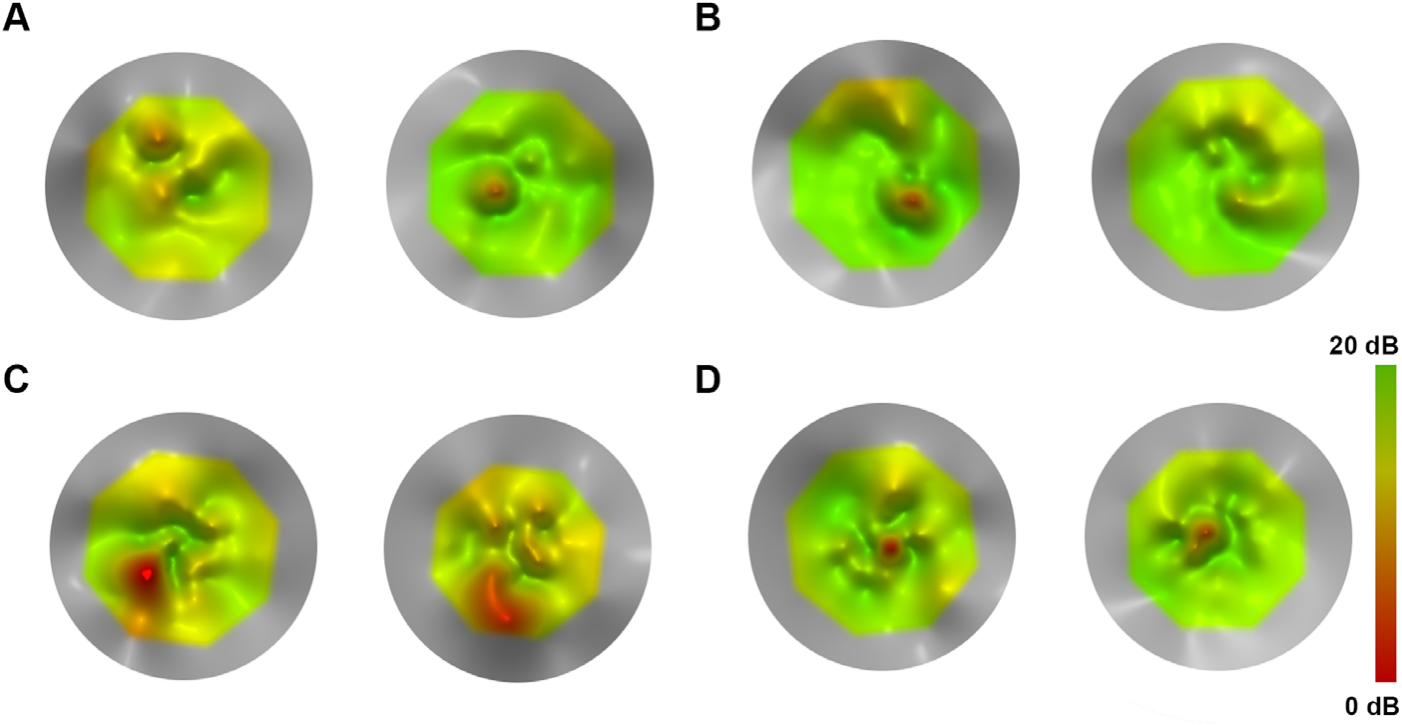
Topographical models using MP-1 microperimetry data of tests performed longitudinally (*left plot* for baseline test and *right* *plot* for follow-up test) on the right eyes of four patients to assess disease progression. (**A**) A 33-year-old female with *CNGB3*-ACHM (MM_0022) with an increase of MS beyond the repeatability coefficient (2.85 dB) over 6.7 years of follow-up. (**B**) A 19-year-old female with *ATF6*-ACHM (MM_0147) with a minimal difference (0.1 dB) over 7.8 years. (**C**) A 7-year-old male with *CNGA3*-ACHM (MM_0165) with the greatest difference in the cohort (increase by 3.2 dB over 8.1 years). (**D**) A 49-year-old female with *CNGA3*-ACHM (MM_0446) with a small difference in MS (0.6 dB) over 7.3 years.

Mean MS, V_TOT_, and V_5°_ were similar to baseline (mean MS: paired *t*-tests, *t* = 0.531, *df* = 17, *P* = 0.602; V_TOT_: paired *t*-tests, *t* = 0.82, *df* = 17, *P* = 0.424; V_5°_: paired *t*-tests, *t* = 0.919, *df* = 17, *P* = 0.371). Figure 4B shows MS change over time for each individual patient. The mean MS difference was –0.18 dB (range, –2.6 to +3.2 dB), and the mean absolute difference was 1.11 dB (range, 0–3.2 dB). Figure 5 presents examples of disease progression. Three patients had a change in MS greater than the test–retest repeatability (1.65 dB); two of those had an increase in retinal sensitivity of 2.85 dB (*CNGB3*-ACHM MM_0022; Fig. 5A) and 3.2 dB (*CNGA3*-ACHM MM_0165, Fig. 5C). Of note, the latter was the youngest patient in the cohort. The third patient had a decrease of 2.6 dB (*CNGB3*-ACHM MM_0067; not shown). By definition, 5% of the tested subjects would be expected to differ by a greater amount than the repeatability coefficient. Mean BCEA was significantly lower compared with baseline (paired *t*-test, *t* = 2.942, *df* = 17, *P* = 0.009).

### Genotype Function–Structure Relations

The MS values for subjects with *CNGA3* (*n* = 10), *CNGB3* (*n* = 5), *ATF6* (*n* = 2), and *GNAT2* (*n* = 1) were 16.38, 17.69, 18.65, and 15.5 dB, respectively. MS was similar between *CNGA3*- and *CNGB3*-ACHM (*t*-test, *t* = 1.10, *df* = 13, *P* = 0.292). Figure 4B shows how MS changed over time, with a distinct marker for each genotype. Seven patients were allocated to OCT group 1 (presence of EZ), and 11 to group 2 (absence of EZ); MS was similar between the two groups: 16.64 dB and 17.17 dB, respectively (*t*-test, *t* = 0.502, *df* =16, *P* = 0.62). Twelve patients had foveal hypoplasia (67%)—two with group 1 OCT and 10 with group 2 OCT. Baseline MS values for patients with and without foveal hypoplasia were similar (*t*-test, *t* = 0.4572, *df* =16, *P* = 0.6537). Baseline BCVA was significantly worse in patients with foveal hypoplasia (mean, 0.79 logMAR) compared with patients without (mean, 0.91 logMAR) (*t*-test, *t* = 2.439, *df* = 16, *P* = 0.0267).

## Discussion

In this study, we assessed cross-sectional and longitudinal MP-derived retinal sensitivity with both conventional and volumetric indices of retinal function in a molecularly confirmed ACHM cohort. Good test–retest repeatability, a high degree of interocular symmetry, and disease stability over a long-term follow-up were demonstrated for the first time, to the best of our knowledge.

A major concern in the design of clinical trials is the identification of robust and repeatable end-points. In a study employing the MP-1 device, the repeatability coefficient was 1.81 dB for 50 patients with a range of macular diseases.^36^ In another study that employed the MP-3 device, the calculated repeatability coefficients for healthy subjects and patients with macular diseases were 1.2 dB and 1.6 dB, respectively.^37^ Our study supports a similar overall test–retest repeatability across all ACHM genotypes, with a coefficient of 1.65 dB and no proportional bias between tests, despite the nature of the disease (e.g., poor BCVA, nystagmus). However, it should be noted that, when assessing interocular symmetry, the subject with the greatest difference was the youngest subject in the cohort and also appeared to show an increase in MS greater than the repeatability coefficient at the follow-up visit. At baseline, the eye with the smaller MS was tested first, and a training effect may have led to a greater sensitivity in the subsequent test on the fellow eye. In this 7-year-old patient, only one test was performed at baseline, due to the young age and tiredness. The above case underlines the need for more than one baseline assessment, especially in trials with a pediatric population, and consideration of the assignment of the first test as a training measurement.^38^

ACHM has been targeted by gene therapy trials where the vector is applied monocularly. Previously, we have reported similar BCVA between eyes in *CNGA3*-ACHM (*n* = 31).^16^ Matet et al.^39^ also reported a strong correlation between interocular visual acuities. Our study further supports visual functional symmetry, with similar BCVA, CS, MS, V_TOT_, and V_5_ between eyes. Symmetry of visual function extends to structure in patients with ACHM. Symmetry of OCT structural findings, such as foveal outer nuclear layer thickness and the integrity of the EZ, has also been reported in several studies of ACHM.^15^^,^^18^^,^^20^^,^^26^^,^^28^^,^^40^ Moreover, interocular symmetry extends to the topography of the foveal cone mosaic as imaged with adaptive optics scanning light ophthalmoscopy.^16^^,^^41^ Given the structural and functional symmetry, it can be assumed that both eyes in patients with ACHM have similar therapeutic potential.

Achromatopsia is a clinical diagnosis. The genetic heterogeneity of the disease may lead to a certain variability among groups of patients based on their genotype.^13^^,^^21^ Aboshiha et al.^28^ observed that retinal sensitivity was significantly higher in the *CNGB3* group. In a retrospective study with long-term follow-up, Thiadens et al.^13^ reported that *CNGA3*-ACHM was more severe than *CNGB3*-ACHM. In our cohort, MS was higher for *CNGB3*-ACHM without reaching statistical significance, something that can be attributed to the small number of patients compared with the aforementioned studies. Recently, phenotyping studies in *ATF6*-ACHM reported a severe structural pathology, including foveal maldevelopment and a lack of foveal cones.^11^^,^^42^ Interestingly, the two patients with this genotype (siblings) in our study had retinal sensitivity toward the higher end of the cohort (Figs. 2D, 4B, 5B). In contrast to *ATF6*-ACHM, *GNAT2*-ACHM presents with better preserved retinal architecture.^21^ However, the subject tested in our study had a MS toward the lower end of the spectrum (Figs. 2B, 4B), suggesting a dissociation between structure and function.

A structural feature that can be different among genotypes is foveal hypoplasia*.* All reported cases in the literature with *ATF6*-ACHM have foveal hypoplasia,^7^^,^^11^^,^^42^ all reported cases with *PDE6C*-ACHM and *GNAT2*-ACHM have normal layering of the foveal pit,^21^^,^^26^^,^^43^ and 60% to 70% of patients with *CNGA3*-ACHM and *CNGB3*-ACHM have foveal hypoplasia.^15^^,^^16^^,^^26^ Recently, it has been suggested that structural grading of foveal hypoplasia may predict future vision in patients with infantile nystagmus (including ACHM).^44^ In our cohort, BCVA was better by six ETDRS letters on average in patients without foveal hypoplasia.

Based on their structural and functional findings with aging, different studies have concluded that ACHM either is stable^24^^,^^25^ or is a progressive disease.^26^^–^^28^ In a cohort of 50 patients with a mean follow-up of 5.2 years, Hirji et al.^20^ identified minimal improvement of BCVA with age which was attributed to the possible improvement of nystagmus with age (known from clinical observations). The current study is based on the same patient population as the aforementioned, with a smaller sample size and extended follow-up. We also identified a minimal statistically significant improvement in BCVA compared with baseline (0.08 logMAR or four ETDRS letters). Previous studies have defined a value of 0.3 logMAR (15 ETDRS letters) as a clinically significant change in BCVA.^35^ Small differences in BCVA in ACHM after treatment should be interpreted with caution and in context, given the presented natural history of the disease. Mean BCEA significantly decreased over time in our cohort, in keeping with the hypothesis of lesser nystagmus with age.^9^^,^^18^ Mean CS, MS, V_TOT_, and V_5°_ were stable, in favor of the stationary disease paradigm. Importantly, summarizing retinal sensitivity across space by a single MS value appears to be too simplistic when examining VFMA two-dimensional plots. Localized depressions or peaks could be identified with VFMA, thereby providing a truer picture of the underlying retinal sensitivity across space and over time (Figs. 2^3^,^4^–5).

### Limitations and Future Directions

Several limitations can be identified in the current study, given the increasing knowledge and experience accumulated in the literature for retinal sensitivity testing and ACHM. The baseline testing was performed on average 7 years ago, with the latest available technology at that time. The dynamic range of the MP-1 is limited to 0 to 20 dB and is susceptible to a ceiling effect. It should be noted that eight of our patients had a MS >18 dB. Newer microperimetry devices have a higher range of stimulation intensities compared with the MP-1 and therefore only a minimal ceiling effect. The testing protocol was based on mesopic assessment of retinal function. Newer approaches with combined mesopic, photopic, and dark-adapted scotopic two-color fundus-controlled perimetry allow for greater specificity of photoreceptor function, such as cone- and rod-specific stimuli (red and cyan, respectively).^45^^,^^46^ Despite this limitation, if overall retinal function deteriorates across time in ACHM, this would be captured. It is of interest for the ongoing therapeutic trials to further investigate the test–retest repeatability in different age groups. For example, the three therapeutic trials for *CNGA3*-ACHM are recruiting patients with three different age eligibility criteria: patients 3 to 15 years of age (NCT03758404), patients older than 6 years of age (NCT02935517), and patients older than 18 years of age (NCT02610582). At baseline, only four of our patients were younger than 16 years of age, so further investigation of test–retest repeatability in adults and children was not performed. Static perimetry may provide new insights into ACHM, as it can be used to characterize peripheral retinal function and offers the opportunity for further localized analysis. In the current study, no pointwise analysis was performed for the investigated parameters, but it would be of value in future investigations.

The largest clinical ACHM study reported in the literature had a cohort of 50 patients and was performed by our group.^20^ A limitation of the current study is the number of recruited patients; from an initial cohort of 40 patients,^26^ we repeated the test in 18 patients after a long follow-up time for various reasons, including testing system availability and maintenance, clinical trial participation, withdrawal from the study, and a dynamic population. Larger molecularly confirmed cohorts will have greater power to investigate any possible genotypic variability of retinal function. Recently, we reported slowly progressive maculopathy as a common feature in *PDE6C*-ACHM.^23^ No data were available for *PDE6C*-ACHM for the current study.

## Conclusions

To the best of our knowledge, this is the first in-depth analysis and long-term longitudinal study of retinal function in ACHM. Highly reproducible assessment of retinal sensitivity, which has been demonstrated in this study, is a prerequisite for its use as a clinical trial end-point. Retinal function, including MS, volumetric indices, and CS, appeared to be stable in our ACHM cohort. Improvement of fixation stability and small changes of BCVA over time may be part of the disease natural history.

## Supplementary Material

Supplement 1
