# Supplementary material for: Long-Term Investigation of Retinal Function in Patients with Achromatopsia
Source: Invest Ophthalmol Vis Sci. 2020 Sep 22;61(11):38. doi: 10.1167/iovs.61.11.38 (PMC7509756; doi:10.1167/iovs.61.11.38)
Supplement: Supplement 1 [file iovs-61-11-38_s001.pdf]

[Online-only material]

1. **eFigure 1: Microperimetry testing grid pattern**
2. **eFigure 2: V_{TOT} and V_{5° , volumetric indices of retinal function**
3. **eTable 1: Demographics and Genetics**

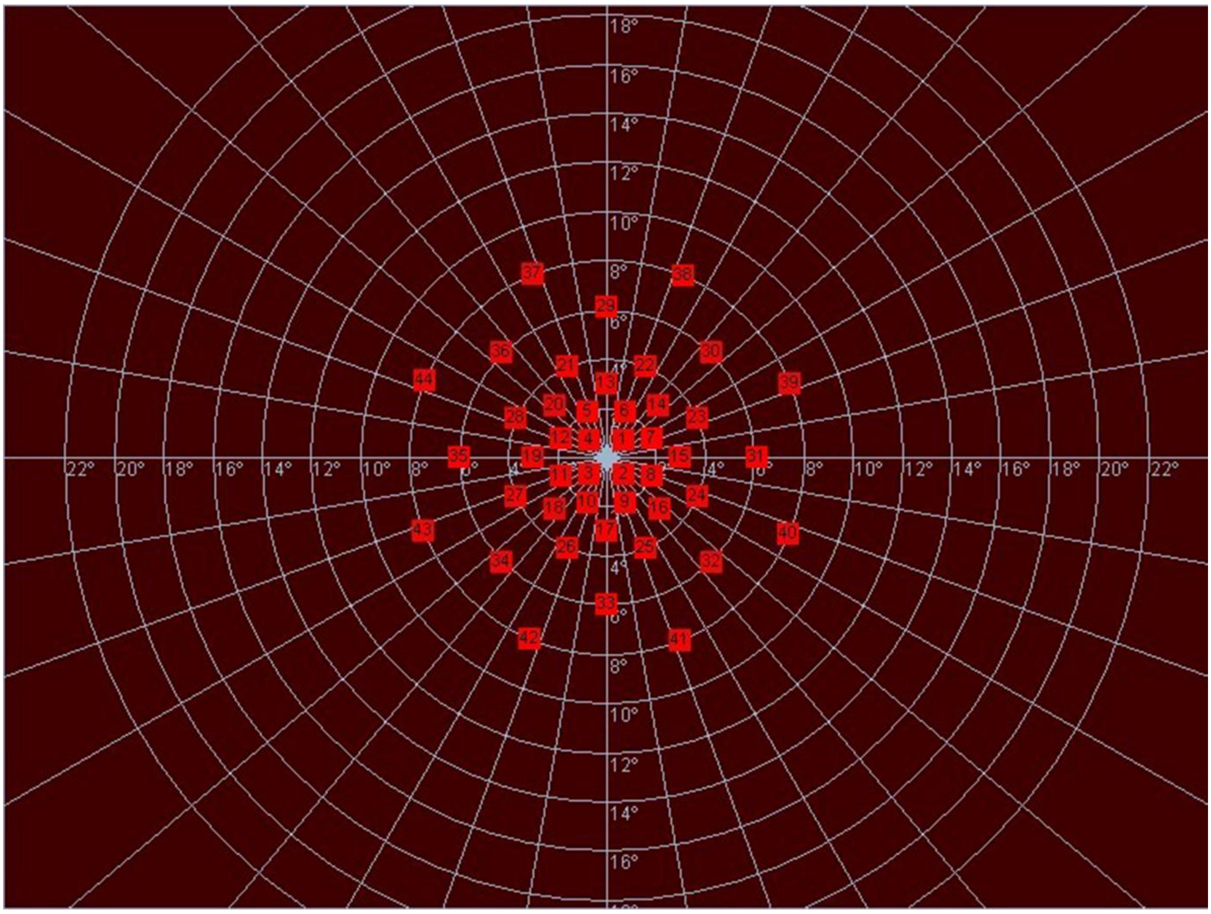

eFigure 1: Microperimetry testing grid pattern.

The customized testing grid consisted of 44 testing locations and 8° radius to cover the macular and paramacular region. The grid pattern was of radial design with centrally-condensed spacing and covered the macular and para-macular region.

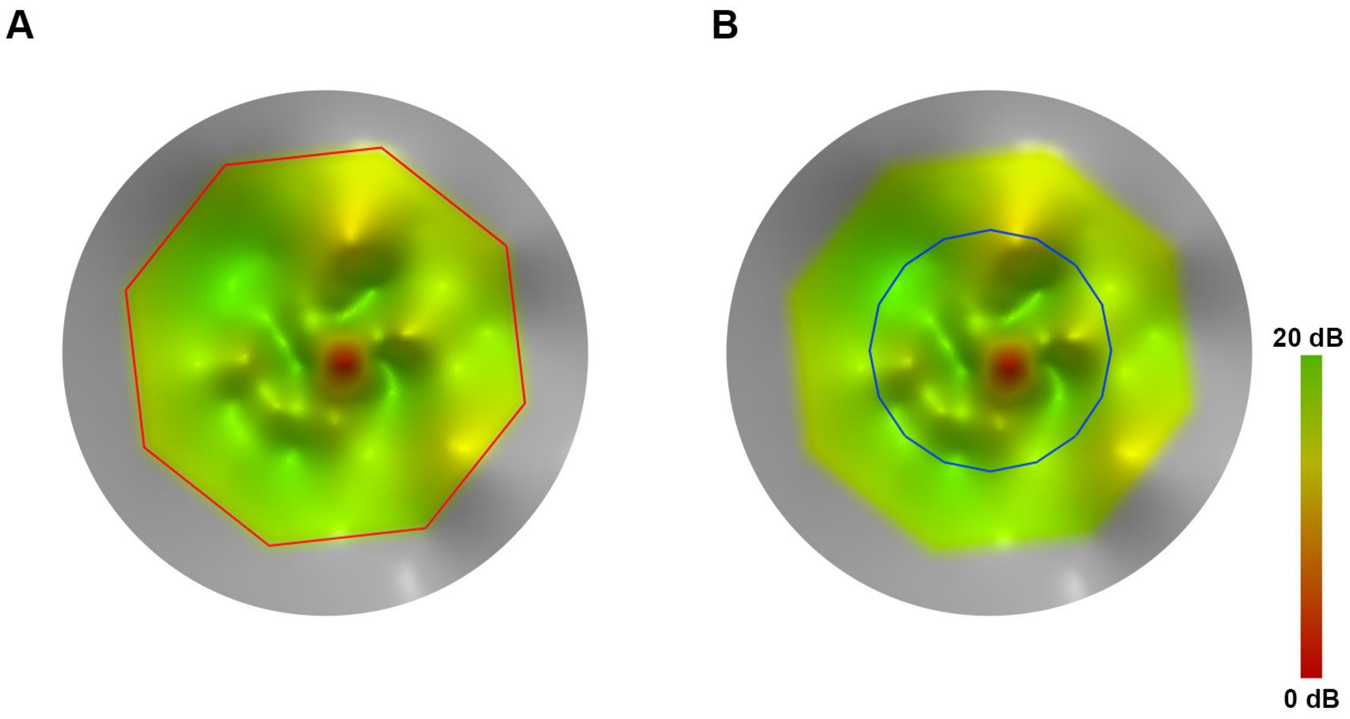

eFigure 2: V_{TOT} and $V_{5°}$ volumetric indices of retinal function

Topographical Model of MP1 microperimetry testing of the right eye, in a subject with *CNGA3*-ACHM (MM_0446). (A) Red outlines the total volume (V_{TOT}), which represented the entire tested field. (B) Blue outlines the central five degrees of the field volume ($V_{5°}$). The volume represents the total sensitivity across the solid angle of the base of the test grid for V_{TOT} and the solid angle of a 5° radius selection for $V_{5°}$, and is reported in units of decibel-steradians (dB-sr). The calibration scale in the figures, reflects the topographic display in sensitivity (dB) from a low of 0 to a ceiling of 20dB.

eTable 1: Demographics, Genetics and OCT Findings

Patient ID*	Sex	Age (years)	Follow-up Time (years)	Hypoplasia	OCT Group§	Gene	cDNA	Protein	cDNA	Protein
MM_0014†	F	29.08	7.58	YES	2	*CNGA3*	848A>G	Arg283Gln	667T>C	Arg223Trp
MM_0015†	F	22.42	6.83	YES	2	*CNGA3*	848A>G	Arg283Gln	667T>C	Arg223Trp
MM_0165	M	7.00	8.08	YES	2	*CNGA3*	1641A>C	Phe547Leu	1641A>C	Phe547Leu
MM_0167	F	22.42	6.58	NO	1	*CNGA3*	847T>C	Arg283Trp	1279T>C	Arg427Cys
MM_0168	M	35.33	7.83	NO	1	*CNGA3*	661T>C	Arg221X	848A>G	Arg283Gln
MM_0169	M	11.08	6.83	NO	1	*CNGA3*	485A>T	Asp162Val	485A>T	Asp162Val
MM_0170	M	10.17	7.50	YES	1	*CNGA3*	1642A>G	Gly548Arg	67T>C	Arg23X
MM_0171	M	17.50	7.25	YES	2	*CNGA3*	1001T>C	Ser334Phe	1360T>A	Lys454X
MM_0446	F	49.67	7.25	YES	2	*CNGA3*	67T>C	Arg23X	67T>C	Arg23X
MM_0480	F	29.33	7.00	NO	1	*CNGA3*	661T>C	Arg221X	848A>G	Arg283Gln
MM_0004	M	23.75	7.25	YES	2	*CNGB3*	1148delC	Thr383Ile fs * 13	1148delC	Thr383Ile fs * 13
MM_0022	F	33.17	6.67	YES	2	*CNGB3*	1148delC	Thr383Ile fs * 13	1148delC	Thr383Ile fs * 13
MM_0067	M	29.83	5.25	YES	1	*CNGB3*	1148delC	Thr383Ile fs * 13	1148delC	Thr383Ile fs * 13
MM_0123	M	47.75	7.42	NO	2	*CNGB3*	1148delC	Thr383Ile fs * 13	1148delC	Thr383Ile fs * 13
MM_0124	M	11.75	6.92	YES	2	*CNGB3*	1148delC	Thr383Ile fs * 13	1148delC	Thr383Ile fs * 13
MM_0147‡	F	19.08	7.83	YES	2	*ATF6*	c.1187+5G>C	p.Asn366His fs*12	c.1187+5G>C	p.Asn366His fs*12
MM_0152‡	F	23.08	7.92	YES	2	*ATF6*	c.1187+5G>C	p.Asn366His fs*12	c.1187+5G>C	p.Asn366His fs*12
MM_0106	M	43.83	7.58	NO	1	*GNAT2*	c.843-844insAGTC	p.His282Ser fs*11	c.843-844insAGTC	p.His282Ser fs*11

*All patients were previously reported[15, 19, 21, 22, 25, 27]

§ OCT Group: Group 1: presence of foveal ellipsoid zone, ii) Group 2: absence of foveal ellipsoid zone.

†‡ Siblings
